# Coherent Terahertz Radiation from Multiple Electron Beams Excitation within a Plasmonic Crystal-like structure

**DOI:** 10.1038/srep41116

**Published:** 2017-01-23

**Authors:** Yaxin Zhang, Yucong Zhou, Yin Gang, Guili Jiang, Ziqiang Yang

**Affiliations:** 1 Terahertz Science and Technology Research Center, School of Physical Electronics, University of Electronic Science and Technology of China, Chengdu 610054, China; 2 National Key Laboratory of Application Specific Integrated Circuit, Hebei Semiconductor Research Institute, China.

## Abstract

Coherent terahertz radiation from multiple electron beams excitation within a plasmonic crystal-like structure (a three-dimensional holes array) which is composed of multiple stacked layers with 3 × 3 subwavelength holes array has been proposed in this paper. It has been found that in the structure the electromagnetic fields in each hole can be coupled with one another to construct a composite mode with strong field intensity. Therefore, the multiple electron beams injection can excite and efficiently interact with such mode. Meanwhile, the coupling among the electron beams is taken place during the interaction so that a very strong coherent terahertz radiation with high electron conversion efficiency can be generated. Furthermore, due to the coupling, the starting current density of this mechanism is much lower than that of traditional electron beam-driven terahertz sources. This multi-beam radiation system may provide a favorable way to combine photonics structure with electronics excitation to generate middle, high power terahertz radiation.

Terahertz (THz) frequency band has been demonstrated of great interest for applications in biomedical imaging, communication, and security checking as the electromagnetic (EM) waves have various physical properties merging photonics and electronics^1^,^2^,^3^. The exploitation of these applications progresses slowly at present for the lack of powerful and compact THz sources. THz waves are usually obtained by optical devices^4^,^5^,^6^, solid state electron devices (SSEDs)^7^,^8^,^9^ and vacuum electron devices (VEDs)^10^,^11^,^12^. Although many progresses have been achieved, the output power level so far is still hard to keep up with demand of the applications^11^,^12^. Compared to the optical devices and SSEDs, the VEDs are more favorable to achieve high power since the electron transport medium is vacuum, which is a “perfect material” for high power devices^12^. Among the VEDs, the linear electron beam (e-beam)-driven sources have been actively investigated in the THz region^13^,^14^,^15^,^16^,^17^,^18^,^19^. However, for these radiation sources, with increased working frequency, higher starting current density is required and lower radiated power obtained^17^. Therefore, many efforts in the linear e-beam-driven sources are concentrated on the design of effective interaction structures as well as on the interaction mechanism so as to improve the radiation intensity and to lower the starting current density.

Multi-beam sources are highly promising candidates to reduce the starting current density and to improve the radiated power. Therefore, in the earlier days, multi-beams have been applied in the microwave sources such as klystrons and free-electron lasers (FELs)^20^,^21^,^22^,^23^. Recently, there are some papers concentrating on multiple beam–wave interaction in the THz region. Such as refs 24, 25, 26, 27 suggested two sheet e-beams interaction with mimicking surface plasmons (MSPs); ref. 28 proposed two sheet e-beams interaction within a composite sandwich structure; ref. 29 reported three- and six-beam folded waveguide traveling-wave tube (TWT) operating at fundamental mode; refs 30, 31, 32, 33, 34 presented two and three e-beams interaction with higher order mode; and ref. 35 theoretically investigated wave coupling in multi-beam TWTs. Although strong efforts have been made and some progress has been achieved in this aspect, an effective mechanism for the multi-beam interaction still seems to be a problem, especially for the case of more than 5 e-beams. Nowadays, the successful exploration of the plasmonic crystal^36^,^37^,^38^ may lead to a bright perspective of multi-beam THz radiation sources.

In ref. 16, we have proposed the interaction between a square-shaped e-beam with guiding wave mode in multiple stacked layers with single sub-wavelength holes (MSLS). The most important is that the MSLS can be can be integrated to construct a plasmonic crystal-like structure that can support multi-beam interaction. Moreover, the coupling of the electromagnetic fields may bring an enhancement of the interaction. Therefore, in this paper, we have proposed an efficient way to generate THz wave from the multi-beam interaction in such plasmonic crystal-like structure.

The plasmonic crystal that made of three-dimensional hole-array layers (3DHA) can support MSP waves which give the possibility of electron beam–MSP interaction and can also provide holes array to act as multi-beam channels. Therefore, multi-beams (9-beams) excitation within such structure which could lead to middle and high output power THz radiation has been studied in detail. The results show that such radiation source system leads to a significant improvement with higher efficiency and lower starting current density than traditional e-beam-driven sources.

## Results

### The model and electromagnetic coupling mode

**Figure 1:**
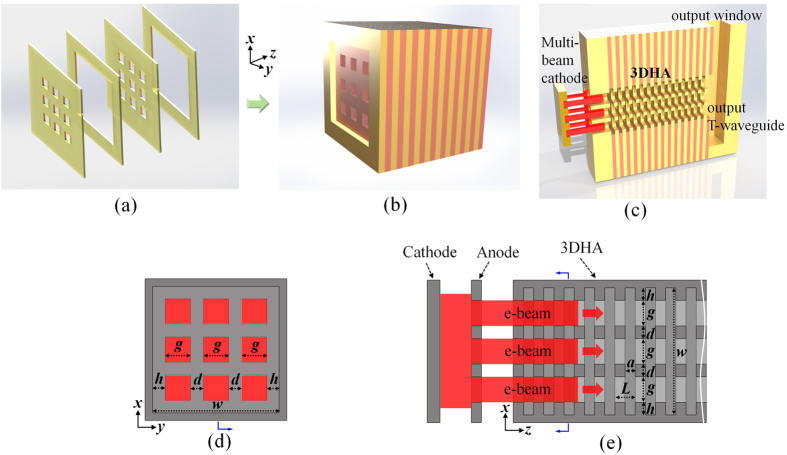
Sketch map of the 3DHA structure. (**a**) Two periodic units of the 3DHA. (**b**) 3DHA consist of several periodic units. (**c**) Metallic modal with multi-beam trajectories (cutaway view) of this source. (**d**,**e**) Cross section and longitudinal section of the 3DHA, respectively.

The 3DHA structure is made of multiple stacked layers with 3 × 3 subwavelength holes array. Figure 1(a) illustrates two periodic units, and each periodic unit is composed of two different hole-layers. One of them is with a large hole and the other is with uniformly distributed 3 × 3 smaller holes array. Each layer can be fabricated separately with metal such as copper. Then, several periodic units are assembled to form the 3DHA structure (Fig. 1(b)). Figure 1(c) demonstrates a 3-D view of the whole design of this 3DHA THz source. A direct-current (DC) e-beam is extracted from the cathode to the anode on which centered 3 × 3 holes array is constructed. Some electrons are intercepted by the anode while the others pass through the 3 × 3 holes array on it to form 3 × 3 multi-beam array and then pass through the 3DHA as shown in Fig. 1(c,e). The cross-sectional and longitudinal views of this structure with multi-beam trajectories and dimensional parameters are shown in Fig. 1(d,e), respectively. It can be found that each hole of the 3DHA acts as an e-beam channel where the injected e-beam passes through and interacts with the MSP wave in the structure.

As we know, for the coherent radiation from the beam–wave interaction, the field intensity and distribution in the structure are key factors. Firstly, the EM field distribution in the 3DHA has been investigated by applying the finite-integral-technique (FIT) eigenmode solver in CST Microwave Studio^39^.

**Figure 2:**
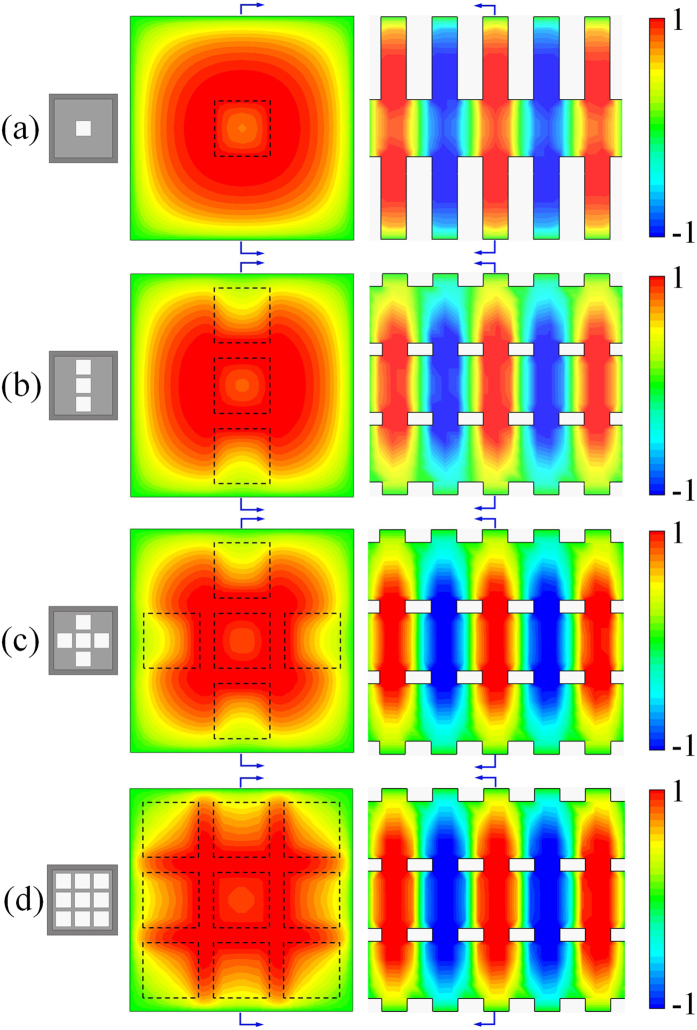
The simulated cross-sectional (left) and longitudinal (right) *E*_*z*_ field distributions of the waves in structures with different holes. (**a**) 1-hole structure. (**b**) 3-hole structure. (**c**) 5-hole structure. (**d**) 9-hole structure (3DHA).

Figure 2 demonstrates that the field distributions in different hole-array structures, where the boundary condition is perfect electric conductor (PEC). For the 1-hole structure in Fig. 2(a), the field distribution is regular around the structure. With the number of holes increasing, the field distribution has been changed as shown in Fig. 2(b–d). Due to the wave coupling among the holes, the distortion of the field takes place. On the other hand, in the longitudinal section, the contour map of the field distribution demonstrates that the mode in the 3DHA has the same EM characteristics of surface plasmons. However, due to the coupling in the center, the mode is not an evanescent wave so that this mode is just a mimicking surface plasmon wave.

**Figure 3:**
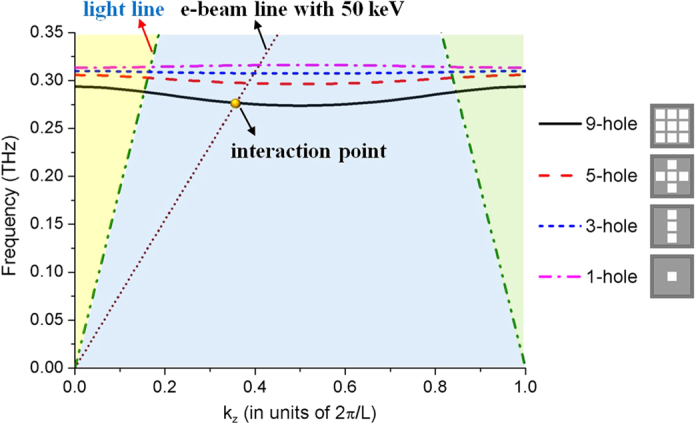
Mechanism of the interaction in the 3DHA system and dispersions of the fundamental modes in different hole-array structures calculated by CST Microwave Studio.

The dispersion relation further shows the EM characteristics of this mode. It can be found from Fig. 3 that with the number of holes decreasing, the upper and cutoff frequencies increase and the dispersion passband becomes narrower. It should also be noted that, since the wave can be coupled through the holes array, 1st spatial harmonics of the dispersion curves in 3 × 3 hole-array structures are backward waves while it is forward wave for the 1-hole structure.

**Figure 4:**
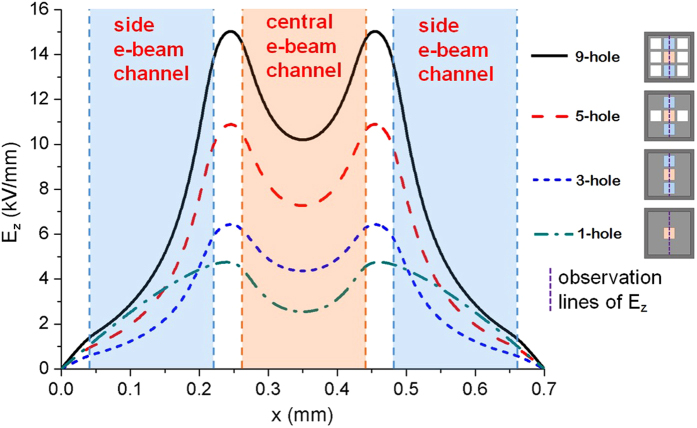
Comparison of amplitude of electric field *Ez* among the coupling modes in the 3DHA, 5-hole, 3-hole, and 1-hole structures.

Moreover, by comparing the contour map of the field in Fig. 2, it is shown that the coupling has enhanced the field intensity. Next, we have undertaken a comparison of amplitude of the longitudinal electric field (*E*_*z*_) among the modes in the 3DHA, 5-hole, 3-hole, and 1-hole structures under PEC boundary condition by a 3D finite-difference time-domain (FDTD) simulation^40^. From the comparison result shown in Fig. 4, it can be found that the field intensities of the mode in the 3DHA are much stronger than those in the rest structures. As we know, in the beam–wave interaction, the intensity of *E*_*z*_ at the e-beam location determines the interaction efficiency and radiation intensity. Thus, it can be expected that the multiple beam–MSP wave interaction in the 3DHA structure may be quite favorable.

### Multi-beam interaction in 3DHA

We now analyze the multi-beam excitation and interaction with such mode in 3DHA system. First of all, when the longitudinal direct-current (DC) e-beam is injected in the modulation area, the fundamental mode of the structure will be excited. The synchronization and interaction occurs when the phase velocity of the mode matches the velocity of the e-beam. During the interaction, the DC e-beams exchange energy with the mode so the velocity and density of the DC e-beams will be modulated and the DC e-beams be bunched. Figure 3 illustrates the mechanism of the interaction. The synchronization condition is fulfilled *k*_*z*_ = *ω*/*v*_*z*_, where *v*_*z*_ is the beam velocity. The intersection between the e-beam line and the dispersion curve of the mode in the 3DHA (the interaction point shown in Fig. 3) is just fulfilled the condition. Therefore the interaction frequency is determined by the dimensional parameters and the beam voltage. The parameters of the system are listed in the Table 1.

**Figure 5:**
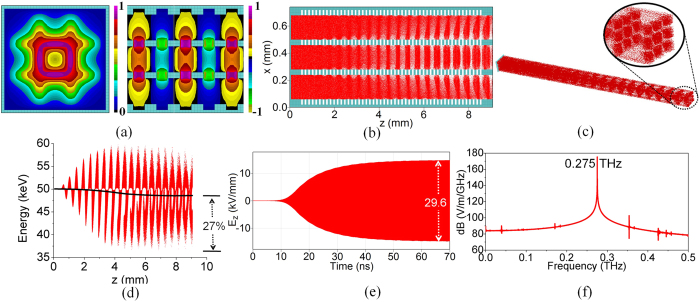
Simulation results of multiple beam–MSP wave interaction in the 3DHA. (**a**) The contour maps of electrical field *E*_*z*_ (normalized) at the cross section (left) and longitudinal section (right). The phase space of the multi-beams in profile (**b**) and 3-D view (**c**). (**d**) The energy distribution and average energy of the multi-beams. (**e**,**f**) The time domain waveform of *E*_*z*_ and its frequency spectrum.

A 3D simulation has been performed with a fully EM particle-in-cell (PIC) code CHIPIC^41^ based on the FDTD method applying the parameters listed in Table 1 to simulate the interaction. The 3 × 3 multi-beam array pass through the structure to stimulate the EM mode. In Fig. 5(a), the special coupling mode is clearly excited and the field distribution is almost the same as the analysis of the eigenmode without the e-beams in Fig. 2(d). Figure 5(b,c) demonstrate the phase space of the multi-beams in profile and 3-D view, respectively. During the interaction, the DC multi-beams are found to synchronize and interact efficiently with the coupling mode and then are gradually modulated and well bunched. It can be observed that in Fig. 5(d) the e-beam exchange the energy with the mode efficiently so that the modulation depth which is directly related to the interaction efficiency can reach near 30%. Figure 5(e) shows that the amplitude of *E*_*z*_ in the structure can reach 14.8 kV/mm. Both modulation depth and amplitude of *E*_*z*_ are relative high values for VEDs in the THz region. Figure 5(f) presents the frequency spectrum, fast Fourier transformed from the time domain waveform (Fig. 5(e)). The interaction frequency 0.275 THz agrees well with the dispersion relation portrayed in Fig. 3. More important, the operating current density of the interaction is only 6 A/cm^2^ which is a fairly low value for VEDs in the THz region.

**Figure 6:**
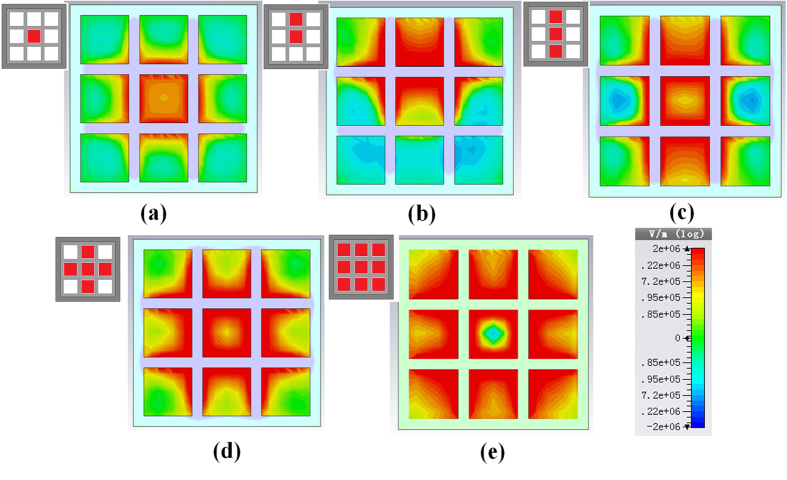
The field intensity distribution of the space charge wave with different numbers of e-beams. (**a**) the one-beam interaction. (**b**) The 2-beam interaction. (**c**) The 3-beam interaction. (**d**) the 5-beam interaction. (**e**) the 9-beam interaction).

Next, we have studied the coupling among the e-beams. The simulation results are shown as below. The Fig. 6 is the field intensity of the space charge wave field of the e-beam. The colors describe the field intensity, the deeper the larger. It can be found that for one e-beam as shown in Fig. 6(a), the e-beam just interact with the mode of its own hole. Therefore, the space charge field is not very strong and concentrates around the hole. It should be noted that one period of this structure is composed of two layers, the one is with a large hole and the other is with 3 × 3 holes array. Therefore, in the 2-beam interaction shown in Fig. 6(b), when the beams pass the larger hole-layer, they meet with each other face to face. Then the space charge waves of both e-beams couple with each other through the hole so that the field intensity has been improved. It can be observed that at the border between the two beams, the field has enhanced significantly. With the number of e-beam increased, the coupling intensity is enhanced. For the 3-beam and 5-beam interaction, the Fig. 6(c,d) demonstrated that hence the center beam couple with beside beams the field intensity at the edge becomes larger and the size of the strong field location also has been enlarged. As a result, as shown in Fig. 6(e), due to the strong coupling among the 9-beam, the field intensity become much higher and such field covers larger region.

**Figure 7:**
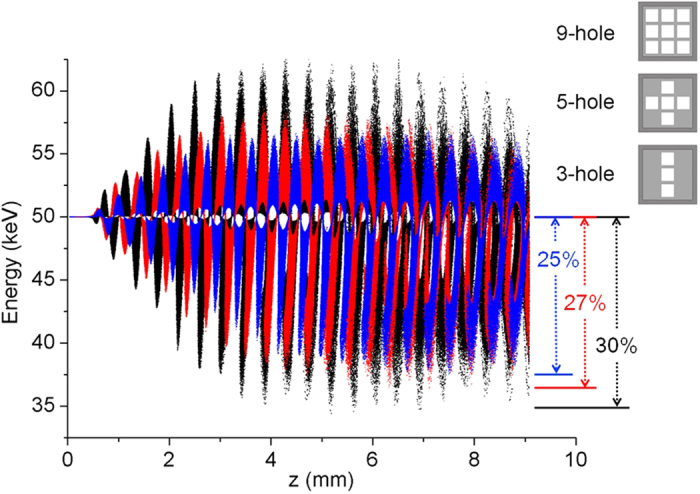
Comparison of modulation depth of the multi-beams among the interactions in 3DHA, 5-hole and 3-hole structures with the same current density of the multi-beams around 0.28 THz working frequency.

Moreover, the space charge wave in the e-beam directly corresponds to the modulation and efficiency. Thus, in order to further illustrate the role of coupling in the multi-beams–3DHA interaction, different number of holes and e-beams interaction have been applied in the simulation as shown in Fig. 7. It is clear that with the beam-number decreasing the modulation depth is becoming smaller. Such results show that the coupling among e-beams can enhance the interaction efficiency to improve the modulation depth.

**Figure 8:**
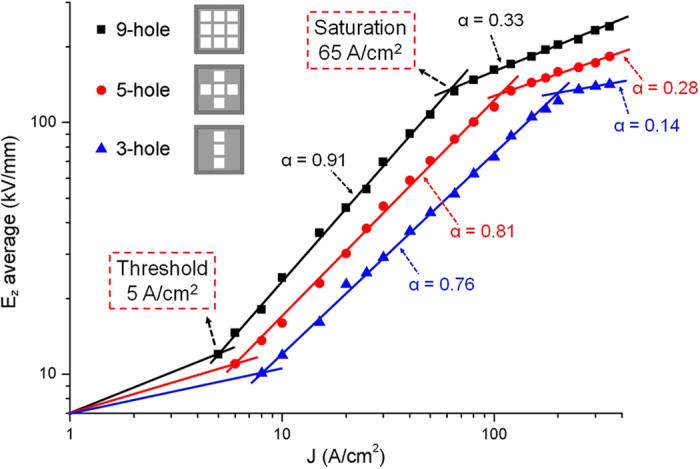
Radiation intensity versus beam current density in the 3DHA (9-beam), 5-hole (5-beam) and 3-hole (3-beam) structures simulated by CHIPIC. Fits were made of the form *y* = *Ax*^*α*^.

It is known that the coherent radiation requires high current density (generally > 30 A/cm^2^) for the VEDs in THz frequency band^11^, which is a key factor limiting developments of the e-beam-driven THz oscillator-sources. In this radiation system, the starting current density is considerably low. In a 50-period 3DHA structure, fixing the beam energy at 50 keV, we sweep the beam current density. The optimized simulation results are shown as black squares in Fig. 7. The region between the threshold and saturation points is the linear growing region where the radiation intensity from the interaction linearly increases with the current density. The starting current density is only 5 A/cm^2^, which is a fairly low value for THz radiation sources, and the saturation point is about 65 A/cm^2^. Besides, we also obtained the optimized radiation intensities as a function of the beam current density in the 5-hole (5-beam) and 3-hole (3-beam) cases, which are respectively illustrated as red circle and blue triangle in Fig. 8. It can be found that the threshold and saturation current density decrease with the number of e-beams increases, which could be considered as the coupling among the multi-beams could enhance the interaction coupling impedance so that the starting current can be reduced.

**Figure 9:**
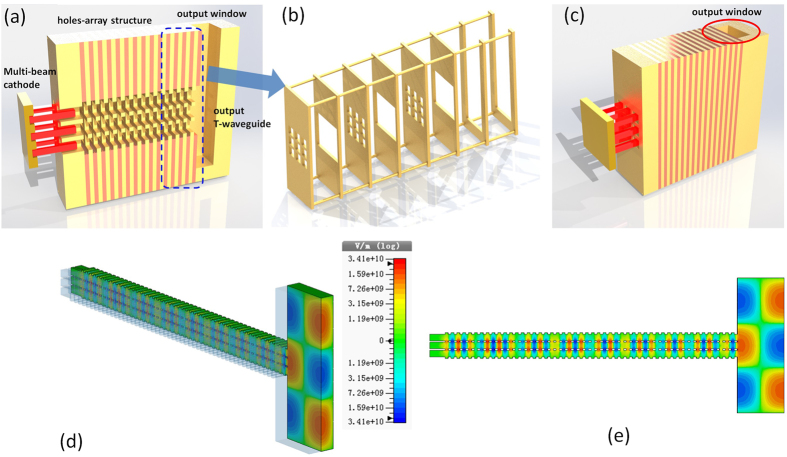
The skech map of the design of the whole system. (**a**) the 3-D view of the cross-section of the whole schematic structure of the system. (**b**) Sketch of assembling (**c**) (**a**) the 3-D view of the the whole schematic structure. (**d**) The 3D contour map of the output field. (**e**) The 2D contour map of the output field.

Next, the output structure has been studied. As shown in Fig. 9(a,b) and Fig. 9(c), for this radiation system the T-coupler waveguide has been applied to output the radiation. The output window is just a standard waveguide port which can connect with an antenna to emit the THz beam. The contour map of the output field is illustrated in Fig. 9(d) and (e). It is clearly that the fundamental mode TE_01_ could be observed.

Moreover, for this structure, the simpler fabrication procedure could be easier than traditional gratings. In general, grating, bi-grating and so on are used the entirely machined technique and the parameters of such structures are always very small at THz, so the microfabrication techniques such as lithographie, galvanoformung und abformung process (LIGA) and deep reactive ion etching (DRIE) have been used. For this structure, it can be made of stacked multiple planar sub-wavelength holes layers. Thus, we can fabricate the holes layers separately and assemble them together to construct the whole structure.

In summary, generation of coherent terahertz radiation from an interaction between multi-electron beam with the coupling mode in a plasmonic crystal-like structure (three-dimensional hole-array structure) which is made of multiple stacked layers with 3 × 3 subwavelength holes array is proposed in this paper. The results show that such multi-beam interaction mechanism can enhance the modulation depth and reduce the current density. Due to multi-beam working, this mechanism can generate high power THz radiation. Moreover, this plasmonic crystal-like structure could provide larger size with more number of holes so that more than 3 × 3 multi-beam array can be applied in this radiation source. At last, this concept of multi-beam excitation within three-dimensional hole-array structure could provide a promising way to develop the compact THz radiation sources with middle or high power.

## Additional Information

**How to cite this article**: Zhang, Y. *et al*. Coherent Terahertz Radiation from Multiple Electron Beams Excitation within a Plasmonic Crystal-like structure. *Sci. Rep.* **7**, 41116; doi: 10.1038/srep41116 (2017).

**Publisher's note:** Springer Nature remains neutral with regard to jurisdictional claims in published maps and institutional affiliations.

## Figures and Tables

**Table 1:** Parameters of the system.

Parameter	Symbol	Value
Period of 3DHA	*L*	160 μm
Number of Periods	*N*	50
Thickness of hole-layer	*a*	80 μm
Side length of larger hole	*w*	700 μm
Side length of smaller hole	*g*	180 μm
Distance between adjacent smaller holes	*d*	40 μm
Distance between larger hole and side smaller hole	*h*	40 μm
Electron kinetic energy	*T*	50 keV
Operating current density	*J*	6 A/cm^2^
Axial guiding magnetic field	*B*	0.5 T
